# A new species of alpine *Apenetretus* Kurnakov from Taiwan: evidences from DNA barcodes and morphological characteristics (Coleoptera, Carabidae, Patrobini)

**DOI:** 10.3897/zookeys.584.6320

**Published:** 2016-04-26

**Authors:** Yi-Ming Weng, Wen-Bin Yeh, Man-Miao Yang

**Affiliations:** 1 Department of Entomology, National Chung Hsing University, No. 250 Kuo-Kuang Road, Taichung, Taiwan 402, R.O.C.; 2 Master Program for Plant Medicine, National Taiwan University, No.1, Sec. 4, Roosevelt Rd., Taipei, Taiwan, 106, R.O.C.

**Keywords:** Apenetretus, Carabidae, Hsueshan, mountain island isolation, new species

## Abstract

There are three isolated mountain ranges in Taiwan including Hsueshan Range, Central Mountain Range, and Yushan Range. The rise of these mountains has resulted in the isolation of some species and caused allopatric distribution resulting in divergence and speciation events of high mountain carabids, especially the flightless carabids such as *Epaphiopsis*, *Apenetretus*, and partial *Nebria*. Genus *Apenetretus* Kurnakov (1960) is typically distributed in high mountain areas of Taiwan. Three of the currently known *Apenetretus* species have been described from different mountain ranges. These species include *Apenetretus yushanensis* Habu, *Apenetretus nanhutanus* Habu, and *Apenetretus smetanai* Zamotajlov and Sciaky. In this study, a new species is described from Hsueshan, a mountain separated from the ranges of the previous known species, *Apenetretus hsueshanensis* **sp. n**. A key to the Taiwanese *Apenetretus* is included. A reconstructed phylogeny of the Taiwanese *Apenetretus* is introduced with the use of mitochondrial cytochrome c oxidase subunit I (COI) gene. Molecular data and geographical distribution of *Apenetretus* support the morphological characteristics observed among those mountain-isolated species and confirms the new species as being distinctly different. Moreover, lineage calibration suggests that the southern *Apenetretus yushanensis* is the most distant one compared to the other three northern *Apenetretus* at ca. 1.81 million years ago (mya), while the divergence time of *Apenetretus hsueshanensis* to its sister group was dated to 0.94 mya.

cytochrome c oxidase subunit I

## Introduction

In Taiwan, mountain ranges that have become isolated over time have played a major role promoting divergent events of high mountain dwelling carabids, especially in species with flightless adults. For example, in *Nebria formosana* Habu and *Nebria niitakana* Kano, morphological variation has been described in populations across mountain ranges (Habu 1972). Ten species of the *Epaphiopsis* Ueno, a genus endemic to Taiwan, are found in the high altitude mountain ranges across Taiwan (Ueno 1989). In addition, the aforementioned species are either allopatrically distributed in specific mountains or have topography-matched divergences. Obviously, these divergent events are highly associated with the effect of mountain isolation.

**Figure 1.**
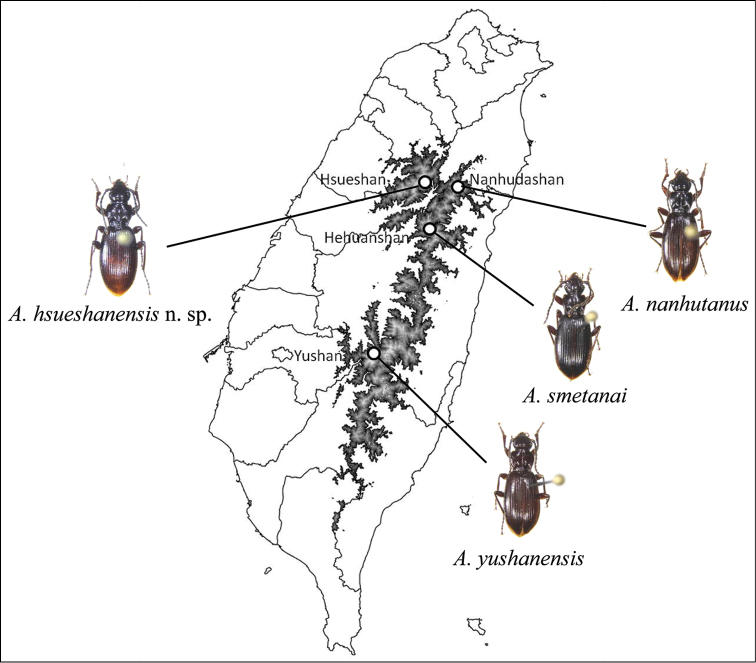
Sample locations of *Apenetretus* spp. *Apenetretus hsueshanensis* sp. n. was collected in Hsueshan; *Apenetretus smetanai* was collected in Hehuanshan; *Apenetretus nanhutanus* was collected in Nanhudashan; *Apenetretus yushanensis* was collected in Yushan. Area of elevation above 2,000 meters is shaded.

The genus *Apenetretus* in Taiwan includes three described species, all of which inhabit alpine areas of different mountain ranges (Habu and Baba 1960; Löbl and Smetana 2003; Terada 2006). *Apenetretus yushanensis* (Habu, 1973) and *Apenetretus nanhutanus* (Habu, 1973) were first collected and described from Yushan and Nanhudashan, respectively (Habu 1973) (Fig. 1). A third species, *Apenetretus smetanai* (Zamotajlov & Sciaky, 1996), was collected by A. Smetana in Mt. Nenggaoshan in 1992. In the original description, *Apenetretus yushanensis* and *Apenetretus nanhutanus* were considered as members of *Patrobus* Dejean, 1821 and *Apatrobus* was considered a subgenus under *Patrobus*. In 1992, Zamotajlov proposed that *Apatrobus* be given genus status with the rationale that members of *Apatrobus* had both larger eyes and more prominent temples which were sub-equal in length with eyes and therefore distinctly different from species of *Patrobus*. Therefore, based on this definition, *Apenetretus yushanensis* and *Apenetretus nanhutanus* are moved from the genus *Patrobus* to *Apatrobus*, and the third species, *Apenetretus semetanai* (Zamotajlov & Sciaky, 1996), was published as *Apatrobus semetanai* as well. Subsequently, the phylogeny among taxa including genus *Apatrobus* was studied and the taxonomy of *Apatrobus* was rearranged accordingly (Zamotajlov 2002; Zamotajlov and Wrase 2006). Two subgenera of *Apenetretus* and *Parapatrobus* are apparently different from *Apatrobus* by the absence of setae on ventral side of claw segments thus the both genera were proposed to new sense as genera. The three species originally belong to the subgenus *Apenetretus* in Taiwan were consequently changed into *Apenetretus yushanensis*, *Apenetretus nanhutanus*, and *Apenetretus smetanai*, respectively (Zamotajlov 2002). Although Lorenz still treated *Apentetretus* as a subgenus of *Apatrobus* in the recent catalog (Lorenz 2005), we expediently follow the classification of Zamotajlov, using *Apenetretus* as the genus for the four species in this study.

According to Habu’s original description, one additional female specimen with larger body and longer, depressed elytra from Mt. Hsueshan (Mt. T’zu-kao) has been collected and was considered by him as a local variety of *Apenetretus yushanensis* (Habu 1973). As more specimens were collected, however, we found several stable characters, including male genital characters, which could be used to distinguish the Hsueshan specimens from the other *Apenetretus* species.

In order to further examine the morphologically similar species, molecular barcoding methods were utilized as a practical process to help reveal candidate cryptic species among numerous unidentified taxa (Burns et al. 2008; Hebert et al. 2003b; Winterbottom et al. 2014; Yassin et al. 2008). Molecular clock method was also employed to analyze *Apenetretus* genetic divergence times. Here the morphological features of a new *Apenetretus* species are described, including a proposed phylogenetic relationship and divergence time with other species based on mitochondrial cytochrome c oxidase subunit I (COI) gene.

## Materials and methods

### Study sites and sample collecting

Specimens of *Apenetretus* were collected by hand from various alpine areas across Taiwan. Specimens from the three species preciously described were collected from their respective mountain ranges including *Apenetretus yushanensis* from Yushan, *Apenetretus nanhutanus* from Nanhudashan, and *Apenetretus smetanai* from near Nenggaoshan (Table 1; Fig. 1). Twenty one individuals of the new *Apenetretus* species were sampled from Hsueshan in stands of Taiwan white fir (*Abies kawakamii*) forest or along brooks near Sanliujiu cabin (ca. 3,330 m). Eight individuals of *Apenetretus yushanensis* were collected from Laonong river campsite (ca. 3,369 m) close to Yushan, twenty *Apenetretus nanhutanus* along the stream in Nanhu glacial cirque, (ca. 3,394 m) near Nanhudashan and fifteen individuals of *Apenetretus smetanai* from the vicinity of Hehuanshan (ca. 3,100 m, close to Nenggaoshan) were collected.

**Table 1.** Sample localities of each species.

	Sample location	latitude	longitude	elevation (m)
*Apenetretus hsueshanensis* sp. n.	Hsueshan	24°23.6N	121°14.7E	3,330
*Apenetretus yushanensis*	Yushan	23°28.5N	120°57.8E	3,369
*Apenetretus nanhutanus*	Nanhutashan	24°22.1N	121°26.5E	3,394
*Apenetretus smetanai*	Hehuanshan	24°8.2N	121°16.5E	3,100

### Morphological measurements

Measurements of morphological characters were done with a Leica S8APO microscope connected to a Canon 600D camera. After taking character photos, images were stacked with software CombineZP (Hadley 2010). Characters were examined and measured with the use of ImageJ 1.48, image analyzing software (Schneider et al. 2012).

### DNA extraction, amplification, and sequencing

For molecular work, twelve individuals of *Apenetretus hsueshanensis* sp. n., ten of *Apenetretus smetanai*, ten of *Apenetretus nanhutanus*, and eight of *Apenetretus yushanensis* were used for DNA extraction. Following the instructions of BuccalAmp^TM^ DNA Extraction Kit (Epicentre Biotechnologies, Madison, WI), genomic DNA was extracted from one hind tarsus of each individual by glass homogenizer grounding in 50 µl QuickExtract Solution, centrifuging for 15 sec, incubating at 65 °C for 10 min, centrifuging for 15 sec again, and then incubating at 98 °C for 2 min. Finally, the resultant genomic DNA products were stored at -20 °C for polymerase chain reaction (PCR).

Mitochondrial COI barcode region was amplified with forward primer Col46 (5’-AACCATAAAGATATTGGAAC-3’) and reverse primer Col731 (5’-CAACAT TTATTTTGATTTTTTGG-3’) in PCR (Tsai et al. 2014). The PCR assay was performed in a volume of 25 µl containing 2 µl genomic DNA extraction as template, 2.5 µl 10X Taq buffer, 0.5 µl Prime Taq DNA polymerase (GENET BIO, Korea), 0.4 µl dNTP (25 µM), and 1 µl of each primer (10 µM). After the initial denaturation at 94 °C for 2 min, PCR programming conditions were followed by 35 cycles of 94 °C for 30 sec, 52 °C for 30 sec and 72 °C for 1 min, with a final extension at 72 °C for 10 min. The PCR products were purified from 1% agarose gel using QIA quick Gel Extraction Kit (Qiagen, Hilden, German). The resulting DNA product was sequenced in both strands using Taq dye terminator Cycle Sequencing Kit (Applied Biosystems, Foster, CA) and an ABI 377A sequencer. Sequences of COI for the four species have been deposited in GenBank under the accession numbers KR868997–KR869036.

### Molecular analyses and phylogeny reconstruction

Sequences were aligned with BioEdit 7.0 software (Hall 1999). Proportional distances among species were conducted using MEGA version 6 (Tamura et al. 2013). The optimal substitution model HKY+I was choice according to jModelTest for Maximum likelihood tree construction and molecular clock calculation (Darriba et al. 2012; Guindon and Gascuel 2003). Phylogenetic inference was performed using maximum likelihood (ML) method with 1,000 bootstrap replications with PhyML version 3.0 (Guindon et al. 2010). The strict molecular clock of the COI gene was calculated under the rate of 3.54% per million years with software BEAST version 1.8.0 (Drummond et al. 2012; Papadopoulou et al. 2010).

## Results and discussion

### Species description

#### Apenetretus hsueshanensis sp. n.

Taxon classificationAnimaliaColeopteraCarabidae

http://zoobank.org/AE24C089-561D-4069-B9E2-422AB3B2E67A

**Figure 2.**
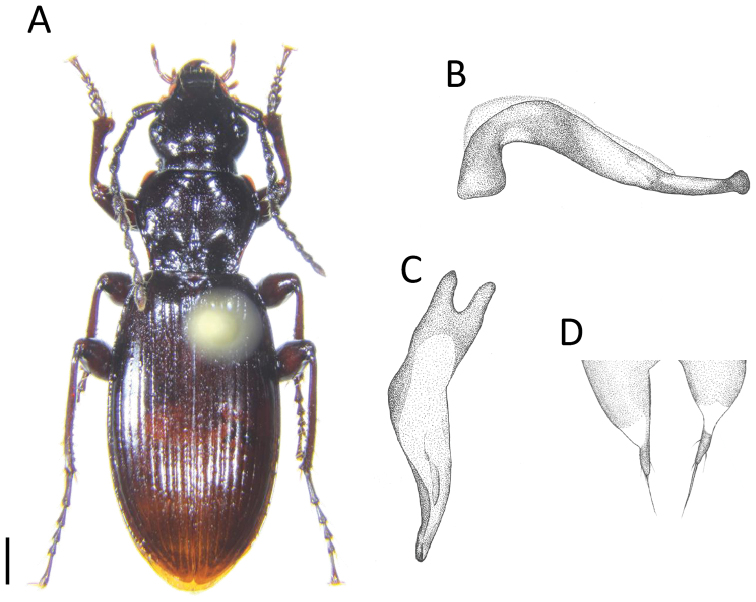
Male of *Apenetretus hsueshanensis* sp. n. (holotype). **A** dorsal view of habitus **B** lateral view of male aedeagus (2×) **C** dorsal view of aedeagus (2×) **D** parameres (3×). Scale bar: 1 mm.

**Figure 3.**
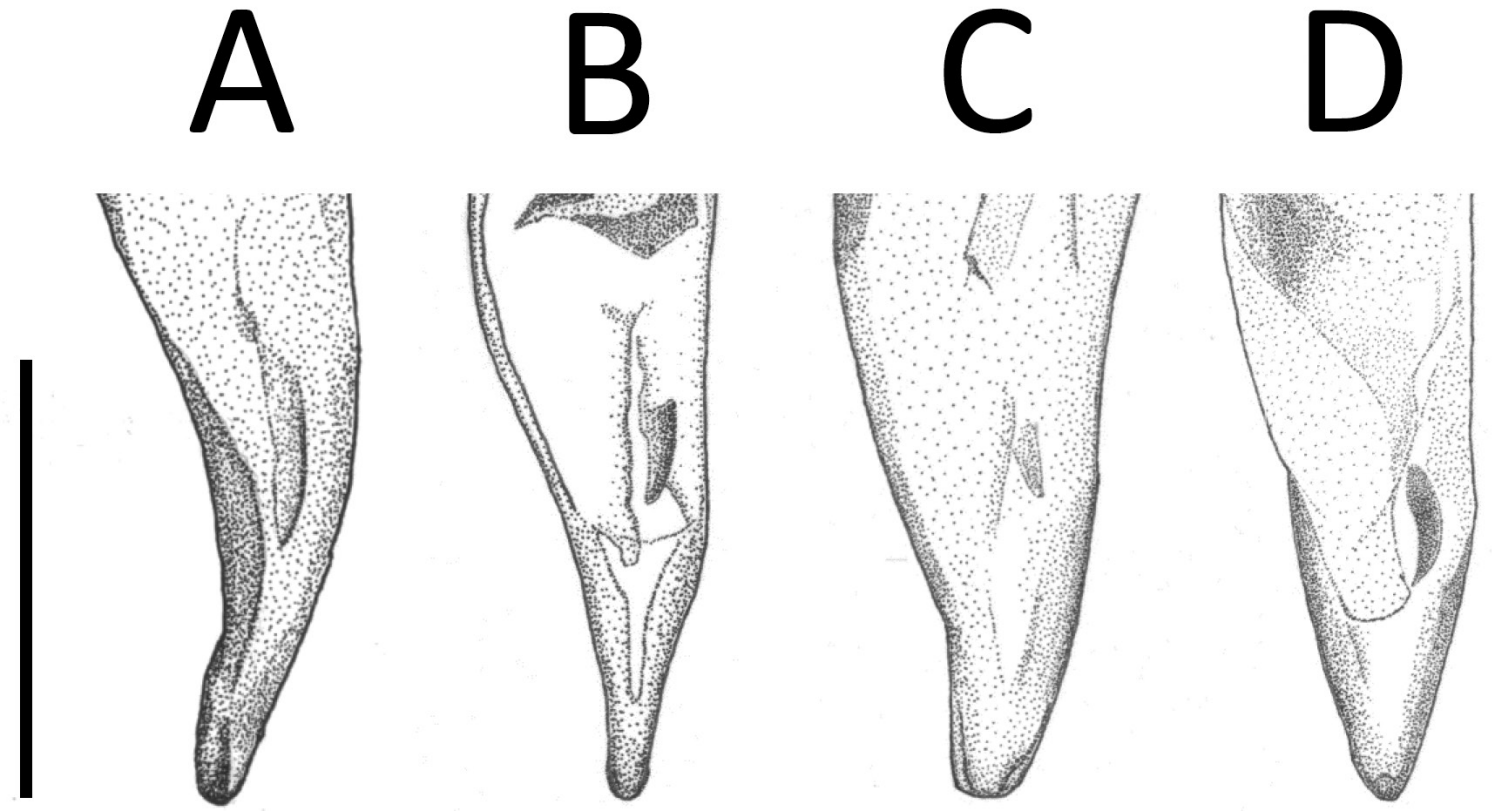
Apical portion of aedeagus of *Apenetretus* spp. in dorsal view. **A** *Apenetretus hsueshanensis* sp. n. holotype **B** *Apenetretus smetanai* **C** *Apenetretus yushanensis* **D** *Apenetretus nanhutanus*. Adapted from Habu 1973; Zamotajlov and Sciaky 1996. Scale bar: 1 mm.

**Figure 4.**
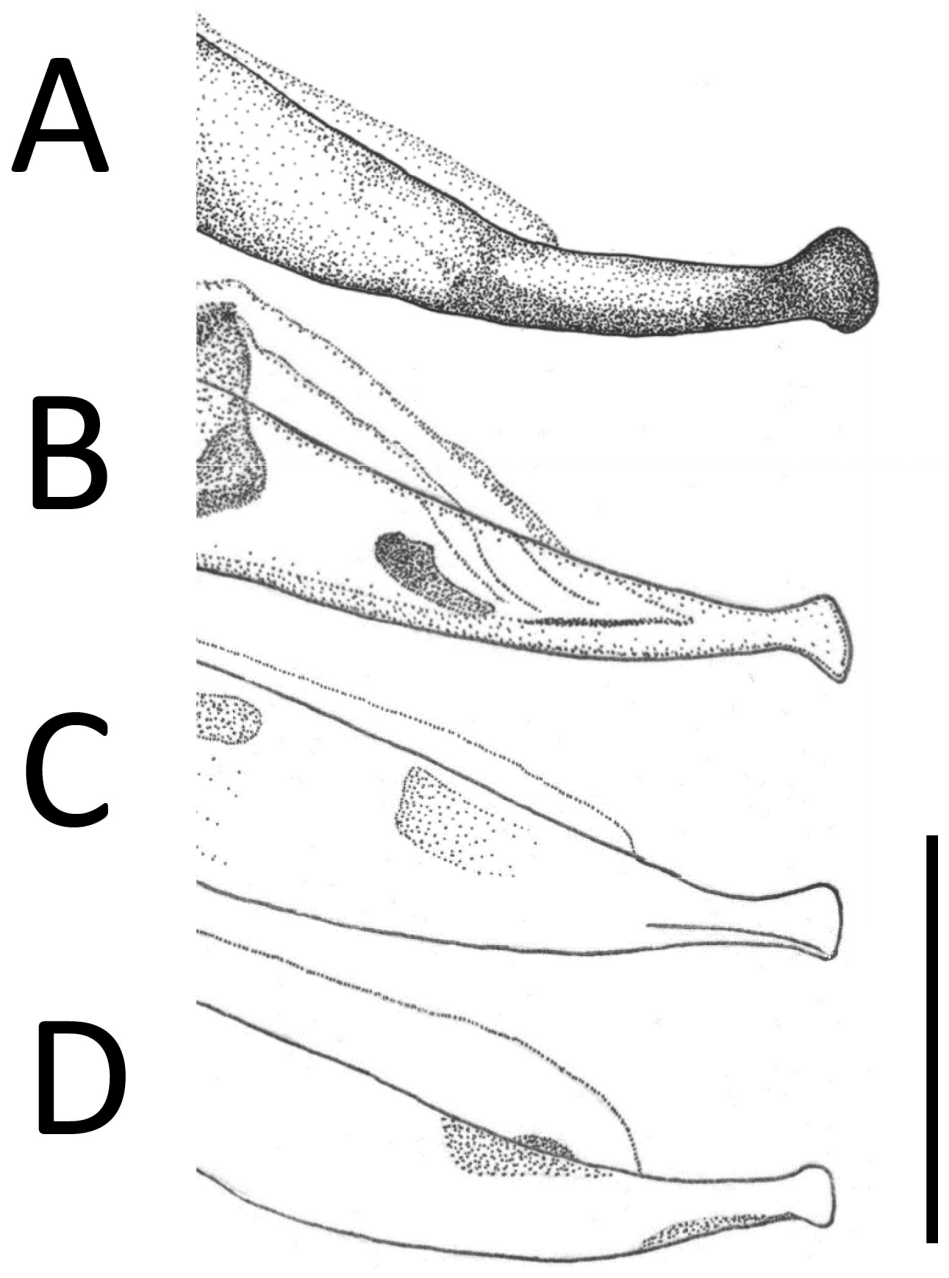
Apical portion of aedeagus of *Apenetretus* spp. in lateral view. **A** *Apenetretus hsueshanensis* sp. n. holotype **B** *Apenetretus smetanai* **C** *Apenetretus yushanensis* **D** *Apenetretus nanhutanus*. Adapted from Habu 1973; Zamotajlov and Sciaky 1996. Scale bar: 1 mm.

**Figure 5.**
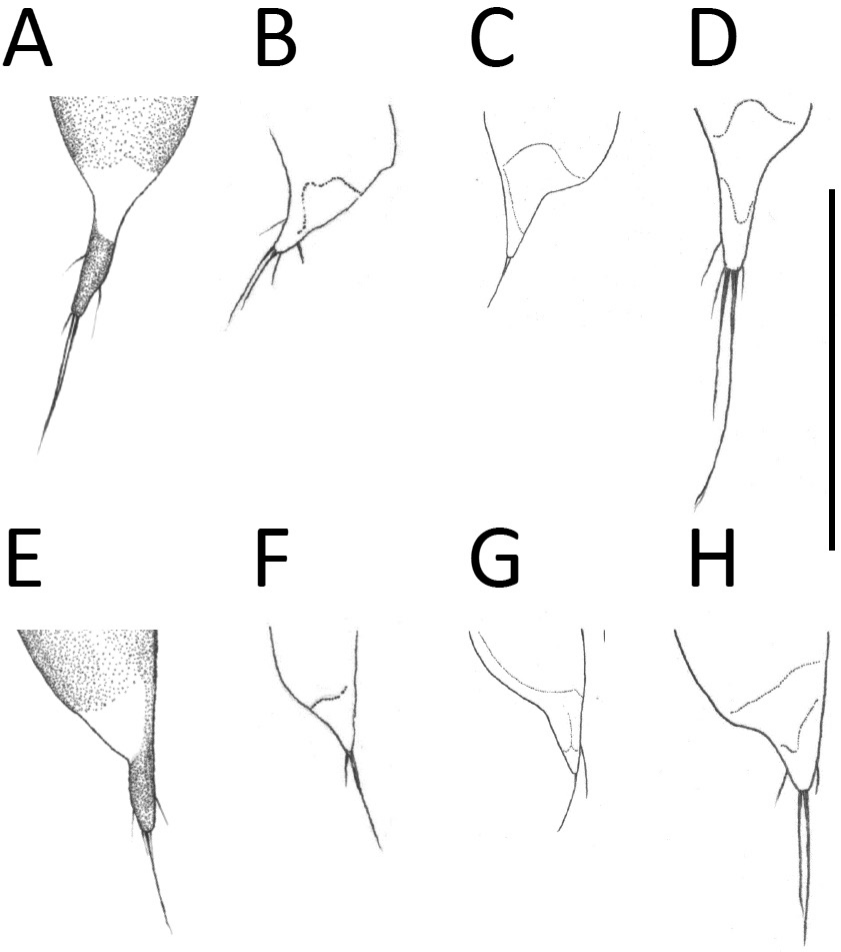
Right parameres (**A**–**D**) and left parameres (**E**–**H**) of *Apenetretus* spp. **A, E** *Apenetretus hsueshanensis* sp. n. holotype **B, F** *Apenetretus smetanai* **C, G** *Apenetretus yushanensis* **D, H** *Apenetretus nanhutanus*. Adapted from Habu 1973; Zamotajlov and Sciaky 1996. Scale bar: 1 mm.

**Figure 6.**
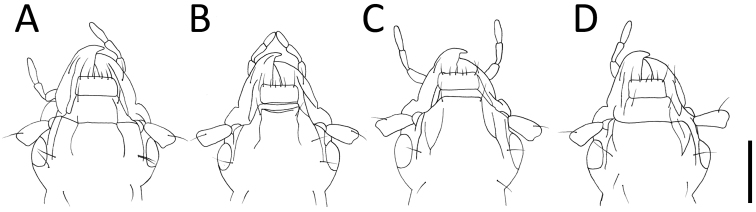
Variation in supraobital setae placement of *Apenetretus hsueshanensis* sp. n. **A** two close anterior setae and one posterior **B** one between eyes and clypeus, one anterior, and one posterior; **C**, one anterior and one posterior **D** one anterior, one between anterior and posterior, and one posterior. Scale bar: 1 mm.

Figs 2 , 3A , 4A , 5A , 5E , 6

##### Type locality.

Taiwan: Mt. Hsueshan, Hsei-Pa National Park, Black Forest near Sanliujiu Cabin, ca. 3,330 m elevation, 24°23.6N, 121°14.7E.

##### Type material.

Holotype: a male, deposited in National Chung-Hsing University (NCHU) Museum of Entomology, labeled: ” TAIWAN, Taichung, Heping District, Hsueshan, Sanliujiu Cabin, 3,330 m, 24°23.6N, 121°14.7E, 08 April 2011, Y. M. Weng collector (red label). Paratypes: A total of 10, 3 males and 4 females with the same collection data as the holotype, 1 male and 2 females labeled: TAIWAN, Taichung, Heping District, Hsueshan, Sanliujiu cabin, 3,330 m, 24°23.6N, 121°14.7E, 01 Oct 2010, Y. M. Weng collector.

##### Etymology.

The new species is named after the original collecting locality, Mt. Hsueshan, where it is likely endemic.

##### Diagnosis.

*Apenetretus hsueshanensis* sp. n. is morphologically similar to the other Taiwanese *Apenetretus* species (*Apenetretus yushanensis*, *Apenetretus nanhutanus*, and *Apenetretus smetanai*). It can be distinguished externally from the other three species by having more slender elytra and a ratio of elytral length/width (EL/EW=1.76–1.90) that differs from all other species (Fig. 2A) 1.67–1.75, 1.67, 1.53–1.67, respectively (Habu 1973; Zamotajlov and Sciaky 1996). This character is especially useful in separating male individuals. Male genitalia; aedeagus large (ca. 3 mm in length) and more slender than the other three species (ca. 2.5 mm in length); extremely elongated and twisted after middle (Figs 3, 4). Apical portion of the parameres is prolonged and longer than the other species (Fig. 5) (Habu 1973; Zamotajlov and Sciaky 1996).

##### Description.

Male 10.79–11.77 mm in length, 3.50–3.79 mm in width, female 11.10–12.22 mm in length, 3.71–4.01 mm in width. Color brown to black, ventral surface reddish brown; labrum, mandibles, palpi, legs, and margin of pronotum and elytra lighter in color (Fig. 2A).

Head convex, frontal impression, neck-constriction punctate; microsculpture faint and isodiametric in dorsal view; neck-constriction deep; temporae faintly tumid, longer than eyes, 1.11 (0.88–1.25) times as long as eye in average (only one individual in fifteen individuals has longer eye length than temporae); eye large, convex; with tooth at subapical terminal; palpi truncate at apex; supraorbital setae varied, some individuals have two closely anterior and one posterior (Fig. 6A), some with only one anterior and one posterior (Fig. 6C), sometimes one between eyes and clypeus, one anterior, and one posterior (Fig. 6B), or one anterior, one between anterior and posterior, and one posterior (Fig. 6D); distance between supraorbital posterior setae rather short, 0.78 (0.73–0.84) times as wide as anterior seta distance; frontal impressions deep, reaching clypeal setae, sometimes divergent posteriorly as *Apenetretus smetanai*; third segment of antenna rather long, 1.47 (1.23–1.59) times as long as forth segment; forth segment of antenna longer than fifth segment, 1.09 (1.03–1.15) times as fifth segment; tenth segment 1.78 (1.68–1.94) times as long as wide; eleventh segment rather prolonged, 2.5 (2.29–2.77) times as long as wide.

Pronotum weakly convex, widest at about one third, 1.22 (1.18–1.32) times as wide as head, 1.23 (1.17–1.29) times as wide as long, 1.35 (1.28–1.39) times as wide as posterior margin, anterior generally as wide as posterior margin, 1.00 (0.95–1.06) times as anterior margin as posterior margin; microsculpture faint and isodiametric; anterior margin straight to rounded and protrudingt at angles; surface faintly punctate at apical areas, rather punctate along median line, lateral margins, and basal area; posterior margin straight, shallowly sinuate near hind angles; hind angles acute to rectangular, slightly prominent laterally; lateral margin subsinuate, from front angles to the widest points, rather round from the widest points to the turning points, then prominent to the posterior seta pore; anterior marginal setae located before the widest point; posterior setae in hind angles; median line deep, sometimes reaching both extremities, generally reaching to anterior transverse impression; anterior transverse impression shallow, sometimes deep and forming a Y-shaped impression; posterior impression and basal foveae deep; disk smooth, rather cordate.

Wings atrophied, 0.3 times as long as elytra; elytra rather convex, ovate and more slender than the other three species (Habu 1973; Zamotajlov and Sciaky 1996), 1.82 (1.76–1.90) times as long as wide, widest behind middle, 1.42 (1.30–1.54) times as wide as pronotum, shoulders with one small tooth on each side, wider than posterior margin of pronotum; microsculpture distinct, isodiametric; lateral margin subsinuate before one third, then rounded, apex elongated subapically; striae rather shallow, sometimes finely punctate; scutellary striole punctate; intervals flat, 3^rd^ interval with 3 pores at 0.22 (0.20–0.26), 0.49 (0.41–0.52), and 0.73 (0.69–0.77) times of elytra length; marginal series composed of 10–12 pores.

Mesepistern, metepistern, and mesostern, lateral of prostern, metasternum, and pregenital sterna 1 with distinct punctures; ventral side of neck constriction shallowly rugose on each side; metepistern longer than wide.

Aedeagus (Fig. 2B, C) slender, curved to right side in dorsal view, curved and elongate before middle (Fig. 2B); apical lamella extremely twisted toward right side, forming a ridge at middle in dorsal view and hammer shape at apex in lateral view (Fig. 2C); left margin reflexed and sinuate in dorsal view; parameres different in shape and size of left and right, left paramere wider than right one, apical projection extended, much longer than the other three species, apex with two long and one short setae, and two short setae at each subapical margin (Fig. 2D).

### Key to *Apenetretus* species of Taiwan

**Table:** 

1	Antenna moniliform, reaching to basal one seventh of elytra; apical part of parameres short, with one short seta at apex and one or no subapical seta (Fig. 5C, G)	***Apenetretus yushanensis* Habu**
–	Antenna slender, reaching to basal one fifth to one sixth of elytra; apical part of parameres longer, with two long seta and one or no short seta at apex, and two short subapical seta on each side (Fig. 5A, E)	**2**
2	Elytra prolonged, more than one and three fourth as long as wide; aedeagus long, (~3mm), extended and extremely twisted toward right side behind middle (Fig. 3A); apical portion of parameres markedly prolonged (Fig. 5A and 5E)	***Apenetretus hsueshanensis* sp. n.**
–	Elytra not prolonged, one and one half to one and three fourth as long as wide; aedeagus shorter, mostly 2–2.5 mm long, evenly contracted toward apex; apical part of parameres less prolonged (Fig. 5B/F and 5D/H)	**3**
3	Palpi truncate and depressed apically; temporae longer than eye; front angles of pronotum stronger projected	***Apenetretus smetanai* Zamotajlov & Sciaky**
–	Palpi not truncate; temporae same length as eye; anterior angles of pronotum weakly projected	***Apenetretus nanhutanus* Habu**

### Genetic differentiation of *Apenetretus* in Taiwan

**Figure 7.**
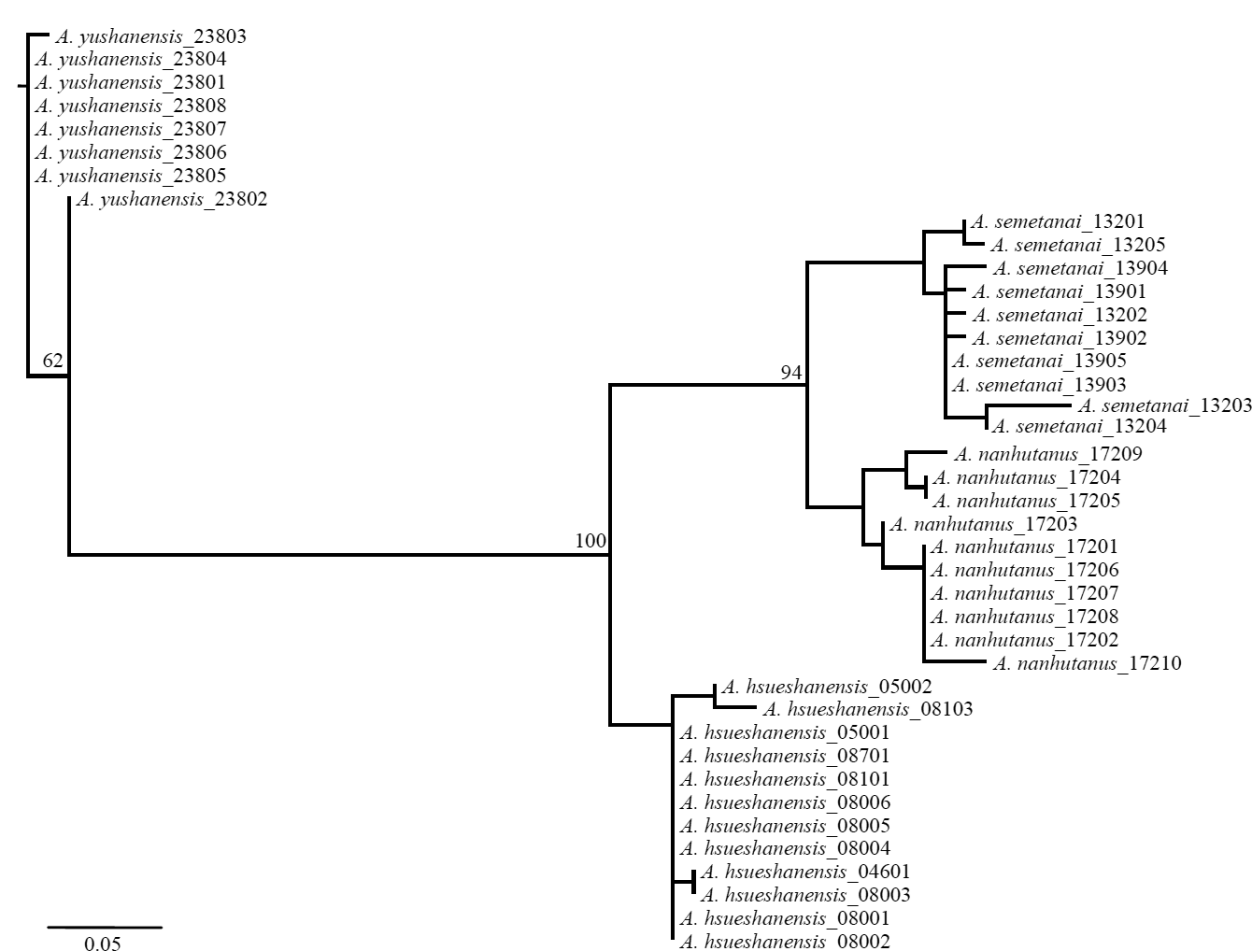
Mitochondrial COI phylogeny of Taiwanese *Apenetretus* constructed with Maximum Likelihood method. One thousand bootstrap values are showed on the branches in percentage.

**Figure 8.**
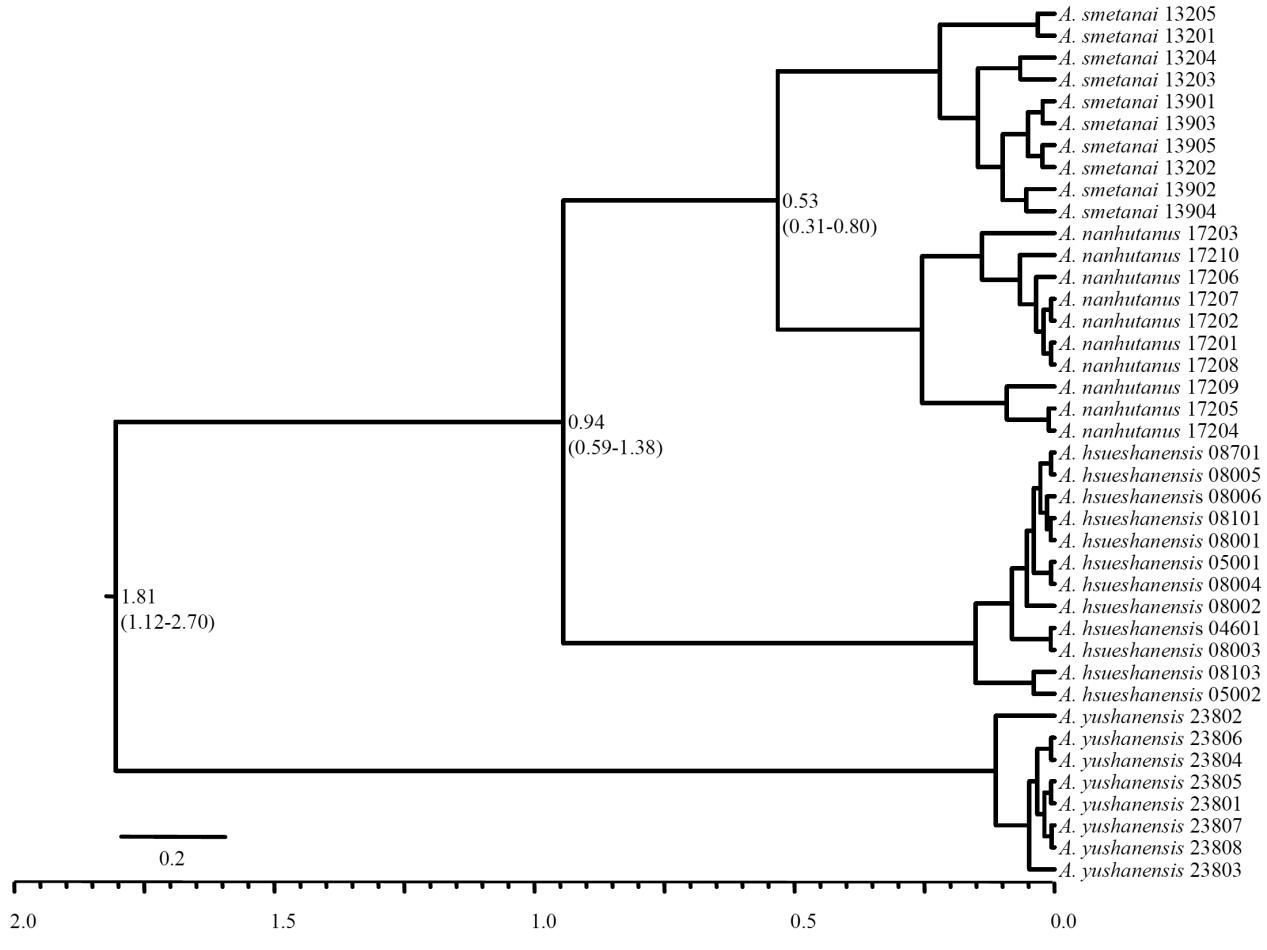
Molecular clock dating of mitochondrial COI gene with BEAST ver. 1.8.0. The oldest divergence between *Apenetretus yushanensis* and the other *Apenetretus* species occurred at 1.81 million years ago (mya); the divergence between *Apenetretus hsueshanensis* and the group of *Apenetretus smetanai* and *Apenetretus nanhutanus* occurred at 0.94 mya; and the divergence between *Apenetretus smetanai* and *Apenetretus nanhutanus* occurred at 0.53 mya.

Phylogenetic analysis of molecular work with the COI gene (686 bp) shows four distinct lineages within the *Apenetretus* of Taiwan (Fig. 7). *Apenetretus yushanensis* is the most basal lineage; members of *Apenetretus hsueshanensis* form a sister group to members of *Apenetretus nanhutanus* and *Apenetretus smetanai* (Fig. 7). The tree topology is consistent with the results of genetic divergence which informs that the most distinct species is *Apenetretus yushanensis* and the least divergent species are *Apenetretus smetanai* and *Apenetretus nanhutanus* (Table 2). It is worth noting that the genetic p-distance among these *Apenetretus* species are close or higher than 2%, the value defined as the general threshold of species differentiation (Hebert et al. 2003a; Hebert et al. 2003b). The divergent trend among *Apenetretus* species is likely to fit with the geological topology of the mountain ranges in Taiwan, where Yushan and Hsueshan Ranges are distinct from Central Mountain Range including Hehuanshan and Nanhudashan. The southern *Apenetretus yushanensis* is the most divergent one to the other three northern *Apenetretus* at ca. 1.81 million years ago (mya). The divergence time between *Apenetretus hsueshanensis* and its sister group was dated to 0.94 mya, a period which is sufficient for speciation to occur (Fig. 8), which further supports our findings that there is an independent species occurring in Hsueshan. Therefore, the localized Hsuehsan carabids with >2% COI divergent content have most likely speciated allopatrically due to the effect of mountain-island isolation. Interestingly, the divergent time between *Apenetretus smetanai* and *Apenetretus nanhutanus*, the two most closely distributed and morphologically similar species appear to have diverged only 0.53 mya. It is yet unclear if there is a geographical barrier between two species, so the possibility is exit that the two species may have other forms of isolated barrier such as isolated by distance or intermittently contact due to glacial cycles. The question can be resolved only by examinations and analyses of series collection along Central Mountain Range.

**Table 2.** P-distance among species of COI gene.

	*Apenetretus hsueshanensis* sp. n.	*Apenetretus smetanai*	*Apenetretus nanhutanus*
*Apenetretus hsueshanensis* sp. n.	-	-	-
*Apenetretus smetanai*	0.027	-	-
*Apenetretus nanhutanus*	0.024	0.019	-
*Apenetretus yushanensis*	0.038	0.049	0.048

## Supplementary Material

XML Treatment for Apenetretus hsueshanensis
